# Eosinophilic Infiltration of the Sino-Atrial Node in Sudden Cardiac Death Caused by Long QT Syndrome

**DOI:** 10.3390/ijms231911666

**Published:** 2022-10-01

**Authors:** Simone Grassi, Oscar Campuzano, Mònica Coll, Francesca Cazzato, Anna Iglesias, Francesco Ausania, Francesca Scarnicci, Georgia Sarquella-Brugada, Josep Brugada, Vincenzo Arena, Antonio Oliva, Ramon Brugada

**Affiliations:** 1 Department of Health Surveillance and Bioethics, Section of Legal Medicine, Fondazione Policlinico A. Gemelli IRCCS, Università Cattolica del Sacro Cuore, 00168 Rome, Italy; 2 Department of Health Sciences, Section of Forensic Medical Sciences, University of Florence, Largo Brambilla 3, 50134 Florence, Italy; 3 Centro de Investigación Biomédica en Red de Enfermedades Cardiovasculares (CIBERCV), 28029 Madrid, Spain; 4 Cardiovascular Genetics Center, Institut d’Investigació Biomèdica Girona (IDIBGI), University of Girona, 17190 Girona, Spain; 5 Medical Science Department, School of Medicine, University of Girona, 17003 Girona, Spain; 6 Department of Diagnostics and Public Health, Section of Forensic Medicine, University of Verona, 37122 Verona, Italy; 7 Pediatric Arrhythmias, Inherited Cardiac Diseases and Sudden Death Unit, Cardiology Department, Sant Joan de Déu Hospital de Barcelona, 08950 Barcelona, Spain; 8 European Reference Network for Rare, Low Prevalence and Complex Diseases of the Heart (ERN GUARD-Heart), 1105 AZ Amsterdam, The Netherlands; 9 Arrítmies Pediàtriques, Cardiologia Genètica i Mort Sobtada, Malalties Cardiovasculars en el Desenvolupament, Institut de Recerca Sant Joan de Déu, Esplugues de Llobregat, 08950 Barcelona, Spain; 10 Arrhythmias Unit, Hospital Clinic, University of Barcelona-IDIBAPS, 08036 Barcelona, Spain; 11 Area of Pathology, Department of Woman and Child Health and Public Health, Fondazione Policlinico Universitario A. Gemelli IRCCS, 00147 Rome, Italy; 12 Cardiology Service, Hospital Josep Trueta, University of Girona, 17007 Girona, Spain

**Keywords:** sudden cardiac death, forensic autopsy, post-mortem genetic testing, eosinophilic inflammatory focus, sino-atrial node

## Abstract

Sudden death is defined as the unexpected death of a healthy person that occurs within the first hour of the onset of symptoms or within 24 h of the victim being last seen alive. In some of these cases, rare deleterious variants of genes associated with inherited cardiac disorders can provide a highly probable explanation for the fatal event. We report the case of a 21-year-old obese woman who lost consciousness suddenly in a public place and was pronounced dead after hospital admission. Clinical autopsy showed an inconclusive gross examination, while in the histopathological analysis an eosinophilic inflammatory focus and interstitial fibrosis in the sino-atrial node were found. Molecular autopsy revealed an intronic variant in the *KCNQ1* gene (c.683 + 5G > A), classified as likely pathogenic for long QT syndrome according to the guidelines provided by the American College of Medical Genetics and Genomics. Therefore, there were many anomalies that could have played a role in the causation of the sudden death, such as the extreme obesity, the cardiac anomalies and the *KNCQ1* variant. This case depicts the difficult interpretation of rare cardiac structural abnormalities in subjects carrying rare variants responsible for inherited arrhythmic disorders and the challenge for the forensic pathologist to make causal inferences in the determinism of the unexpected decease.

## 1. Introduction

Sudden death (SD) is defined as the unexpected death of a healthy person that occurs within the first hour of the onset of symptoms or, if death is unwitnessed, within 24 h of the victim being last seen alive [1,2]. About 85% of all SDs are of cardiac origin and account for about the 15–20% of all deaths [3,4]. The annual reported incidence of sudden cardiac death (SCD) in young people (up to 35 years old) is between 1 and 2 cases per 100,000 [5,6,7]. In those younger than 35 years, cardiomyopathies and channelopathies are the most frequent cardiac causes of SCD [1,8]. Among channelopathies, the most common causes of SCD are long QT syndrome (LQTS), Brugada syndrome (BrS), catecholaminergic polymorphic ventricular tachycardia (CPVT) and early repolarization syndrome (ERS) [3,9,10,11]. Among the predictors of SCD, obesity (a well-established independent risk factor for cardiovascular diseases) has been found to increase arrhythmic risk (i.e., every 5-unit increment in BMI confers a 16% higher risk of SCD) [12,13,14,15,16]. When autopsy and toxicological testing fail to find any anomaly or there are macroscopic and/or microscopic anomalies whose significance is uncertain, molecular autopsy (i.e., post-mortem genetic testing) should be indicated [17,18]. Indeed, as stated by the Asia Pacific Heart Rhythm Society and the Heart Rhythm Society in 2020, molecular autopsy in young victims of autopsy-negative SDs should be considered a “public health priority”, allowing for public health interventions such as the clinical/genetic screening of the first-degree relatives [19]. In this paper, we report a case of SD of a young obese woman in apparent good health. The goal of this paper is to report, to the best of our knowledge, the first case of an eosinophilic infiltrate of the sino-atrial node (SAN) in the context of SD, associated with genetic evidence of LQTS.

## 2. Cases Presentation

### 2.1. Case Report

**Figure 1:**
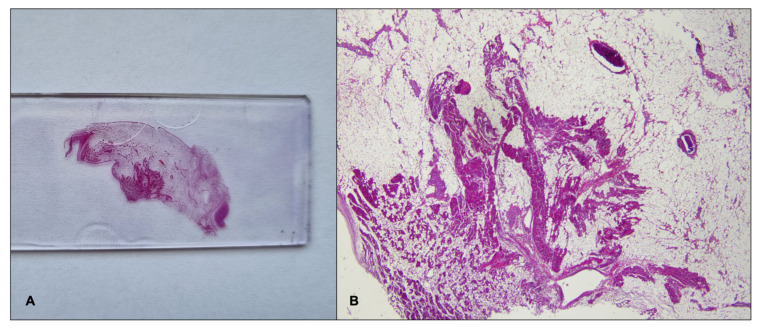
Gross histopathological examination (Hematoxylin and eosin) of the sino-atrial node on whole slide (**A**); (**B**) 1× magnification.

**Figure 2:**
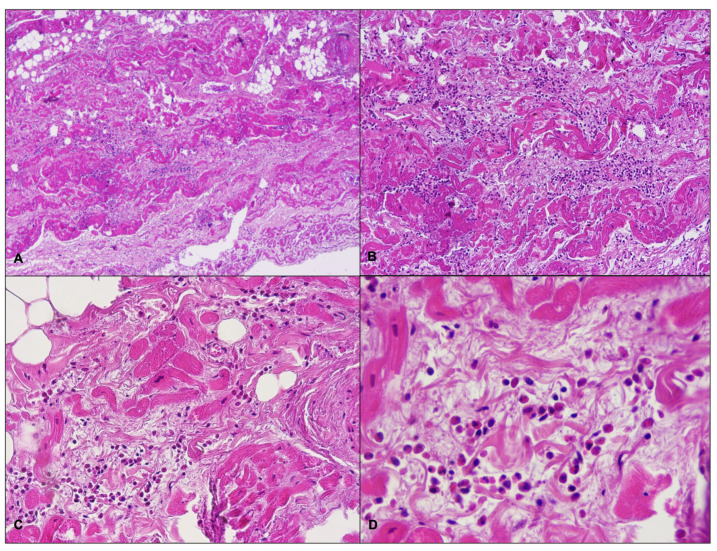
Histopathological examination (hematoxylin and eosin) of the sino-atrial node shows interstitial fibrosis and eosinophilic inflammatory infiltrate: (**A**) 4× magnification; (**B**) 10× magnification; (**C**) 20× magnification; (**D**) 40× magnification.

**Figure 3:**
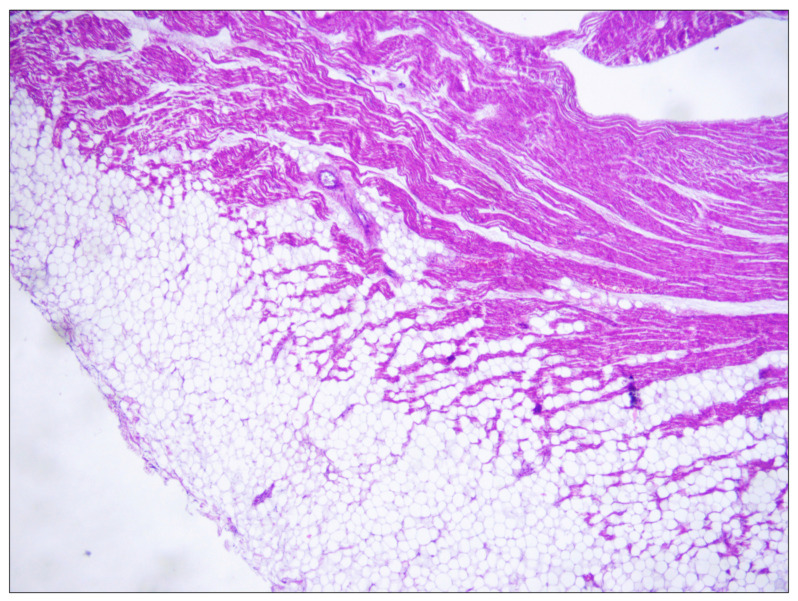
Fatty infiltration separated by the myocardium in the right ventricle wall (i.e., adipositas cordis) (hematoxylin and eosin, 2× magnification).

A 21-year-old obese woman (height: 157 cm; weight: 153 kg; BMI: 62.07 kg/m^2^) suddenly died in a public place while smoking a cigarette. The friend who witnessed her death said that she suddenly lost consciousness without having shown any symptoms previously. The victim’s clinical and family history was negative. Besides significant cardiovascular risk factors such as smoking and obesity, she was in a state of apparent good health: indeed, three months before her death, she underwent a sleeve gastrectomy, and thus pre-operative ECG and biochemical analysis (triglycerides: 119 mg/dL; high-density lipoprotein cholesterol: 43 mg/dL; low-density lipoprotein cholesterol: 94 mg/dL) were performed (with substantially normal results). At hospital admission after the loss of consciousness, she underwent orotracheal intubation and the absence of cardiac electrical activity (asystole) was recorded during ECG monitoring, along with the absence of mechanical cardiac activity identified by echocardiography. Neither pericardial effusion nor altered size of the cardiac chambers were observed. Routine toxicology tests were performed on urine, failing to find traces of the considered substances (opiates, cocaine, cannabinoids, barbiturates, amphetamines and benzodiazepines). After about 60 min of resuscitation maneuvers, the woman was pronounced dead. A full clinical autopsy was requested to find the cause of death, and it was performed three days after the death at the Fondazione Policlinico A. Gemelli IRCCS, Rome, Italy. The medical records did not mention whether she had been vaccinated against SARS-CoV-2, but postmortem microbiological investigations ruled out a SARS-CoV-2 infection. The autopsy did not find any macroscopic anomalies except for a lung edema (lungs weights: left—750 g, right—720 g). The brain and heart were isolated and dissected only after fixation in toto with a 10% buffered formalin-based solution. The gross and histopathological examination of the brain (weight: 1300 g) did not show any relevant findings. During the gross examination of the heart (weight: 350 g, anteroposterior diameter: 5 cm, longitudinal diameter: 11 cm, transverse diameter: 13 cm), only mild right ventricular enlargement was observed. The other cardiac structures and coronary arteries were macroscopically normal. The conduction system was carefully analyzed and serial sectioning targeting blocks of areas of interest was performed in compliance with current guidelines [20,21]. The histopathological examination (hematoxylin and eosin staining) of the myocardium found the wavering of myocardial fibers. During the SAN (Figure 1), an eosinophilic inflammatory focus surrounded by areas of myocardial sclerosis and interstitial fibrosis (in the normal range) was observed (Figure 2). Some eosinophilic infiltrates of the intramyocardial capillary vessels were reported around the SAN and in the left ventricle. The other examined parts of the cardiac conduction system, and in particular the atrioventricular node (AVN), were microscopically normal. The right ventricle wall presented some infiltration of fatty tissue that was separated from the myocardium (Figure 3).

Post-mortem toxicology testing was not requested by the competent authority due to the results of the hospital’s toxicology testing.

### 2.2. Genetic Testing

DNA was extracted from postmortem whole blood with Chemagic MSM I (PerkinElmer, Waltham, MA, USA). Spectrophotometric analysis was performed to evaluate quality ratios of absorbance (260/280:260/230 minimum of 1.8:2.2). DNA concentration was determined using the Qubit fluorometer (Thermo Fisher Scientific, Waltham, MA, USA), and 3 μg of DNA was used for library preparation. NGS analysis was performed using a custom resequencing panel of 85 genes associated with cardiomyopathies and channelopathies, designed and optimized by our group. We sequenced an isoform for each gene and only coding exons and 10 base pairs inside intronic regions. Exons were obtained from Ensembl site in GRCh38 and translated to hg19 [22]. Bait design was performed through an in-house algorithm and submitted to Agilent SureSelect Design Web. Genomic DNA was fragmented through sonication using the Bioruptor (Diagenode, Denville, NJ, US), and the 85 genes were enriched using the SureSelect Custom Target Enrichment System Kit following the manufacturer’s instructions (Agilent Technologies, Santa Clara, CA, USA). The paired-end sequencing process was carried out on MiSeq System (Illumina, Inc., San Diego, CA, USA) using a 2 × 76 bp read length. NGS analysis was submitted to an in-home pipeline [23]. Variant calls for SNVs and small indels were obtained using Samtools (v.1.3.1) and an internal caller. Population data were obtained from the Genome Aggregation Database (gnomAD; http://gnomad.broadinstitute.org, accessed on 13 May 2021). The protein predictors consulted were PolyPhen2 [24], Sift [25], Provean [26] and Mutation Taster [27]. Finally, we also used the splicing predictors MaxEntScan [28], FSPLICE, GeneSplicer [29] and NNsplice [30]. Sanger sequencing was indicated when the coverage was lower than 30X and to validate variants with a frequency lower than 0.1%. Hence, the polymerase chain reaction (PCR) was performed, and after a purification through ExoSAP-IT (USB Corporation, Cleveland, OH, USA), the product was directly sequenced using the dideoxy chain-termination method in an ABI Prism Big Dye^®^ Terminator v3.1 Cycle Sequencing Kit (Applied Biosystems, Waltham, MA, USA). Sequencing was performed using a 3500 Genetic Analyzer (Applied Biosystems, Waltham, MA, USA) and analyzed by SeqScape Software v2.5 (Life Technologies, Waltham, MA, USA). Genetic variants were reported in compliance with the recommendations given by the Human Genome Variation Society (HGVS). All variants were classified as pathogenic (P), likely pathogenic (LP), or variants of unknown significance (VUS) according to standards and guidelines for the interpretation of sequence variants from the American College of Medical Genetics and Genomics (ACMG) and the Association for Molecular Pathology (AMP) [31].

### 2.3. Genetic Results

**Figure 4:**
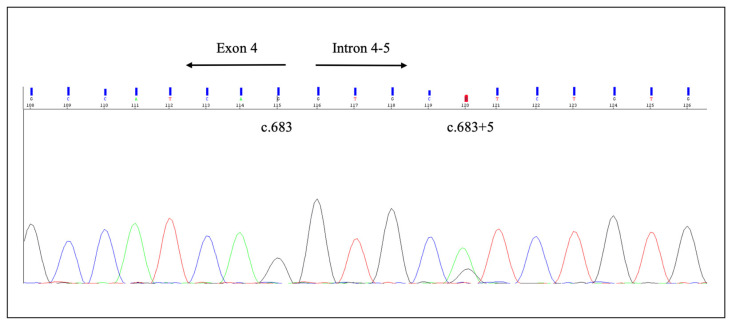
Intronic variant c.683 + 5G > A (rs397508122) in *KCNQ1* gene (NM_000218) identified during molecular autopsy.

Molecular autopsy identified the intronic variant c.683+5G>A (rs397508122) in the *KCNQ1* gene (NM_000218) (Figure 4).

This variant was detected at an extremely low frequency in the general population (GnomAD: allele frequency: 0.0012%; Popmax filtered allele frequency: 0.0002980%). No other rare variants or copy number variants were identified in any of the genes analyzed. This variant was classified as LP following ACMG recommendations, despite discording data in ClinVar. An in vitro splice assay showed that this variant causes exon 4 skipping and premature protein truncation [32]. This variant was reported in the literature in individuals affected with LQTS and Jervell and Lange-Nielsen syndrome [33,34,35,36,37,38,39,40,41]. These reports do not provide unequivocal conclusions about the association of the variant with arrhythmic disorders. In addition, co-occurrence with another pathogenic variant has been reported (*KCNQ1* c.1484_1485delCT, p.L496AfsX19, [41]), providing supporting evidence for a benign role.

## 3. Discussion

We reported the case of the sudden death of a healthy young woman with a clinical history substantially negative but for unspecified previous episodes of acute laryngeal edema of possible allergic origin. During her autopsy, no significant anomalies were found, while during the microscopic examination of the sino-atrial node area, an eosinophilic infiltrate and interstitial fibrosis surrounded by areas of myocardial sclerosis were found. In sudden death victims, it is highly recommended that an expert pathologist carefully examines the cardiac conduction system (CCS), as suggested, among others, by Ottaviani and Buja and by The Royal College of Pathologists [20,21]. In the CCS, there are several anomalies that can be considered relatively common and of no pathologic significance, such as fibrosis of the summit of the interventricular septum, fatty change of the atrioventricular node (AVN), His bundle, or bundle branches, mild to moderate fibromuscular hyperplasia of the SAN or AVN artery and a left-sided bundle of His [42]. That being said, to the best of our knowledge, our paper is the first to report an eosinophilic inflammatory focus of the SAN in a case of sudden death. Bharati and Lev [43] analyzed 14 cases of SDs of athletes, finding that in four of them, an unspecified mononuclear cell infiltration within and around the SAN occurred. They noted that this finding cannot lead to a diagnosis of myocarditis per se, and hypothesized that it may represent a hypersensitivity reaction associated with as yet unexplored immune complexes. The same group reported six cases of SDs of young adults with bronchial asthma, finding unspecified mononuclear cells in the SAN in five out of six cases [44]. Moreover, in 1995 [45], they analyzed seven cases of SDs of young obese persons, finding in all the cases unspecified mononuclear cells within and around the SAN. They hypothesized that some silent arrhythmias could become lethal in susceptible people, such as those genetically predisposed to arrhythmias, those with obesity, those with sleep apnea syndrome or those experiencing altered physiological states. The other finding of the SAN (the fibrosis) is of uncertain significance: although its presence within and around the SAN can be considered physiological (especially in older subjects) and even protective, Grassi et al. reported a case of sudden death in a young person in which one of the few findings was an exuberant representation of fibrosis in this area [1]. Indeed, an upregulation of fibrosis accompanied by replacement of SAN pacemaker cells was found to increase beat-to-beat variability and may lead to arrhythmias and SCD [46,47,48,49]. Hence, according to current evidence, no certain significance can be given to the anomalies found at the CCS [50]. The other finding during the heart microscopic examination (fatty infiltration of the right ventricular wall) is of no pathologic significance, since the disposition of the adipose tissue follows the physiological pattern of the so-called “adipositas cordis” [51]. Indeed, the victim was obese, and fatty infiltration of the heart is a typical and generally benign feature in obese subjects. At macroscopic examination, although the woman was severely obese, her fresh heart weight was unexpectedly low if the significant body weight is considered. The only explanations that can be hypothesized on this matter are related to the woman’s young age (21 years old) and low height (157 cm) [52]. Regarding the actual role of obesity in SCD, in the Framingham Heart Study, the annual mortality rate for SCD in obese subjects was reported to be 40 times higher than in the normal weight population [13,53,54,55]. The development of an arrhythmic substrate has been hypothesized as the predominant underlying mechanism [14]. Some ECG abnormalities, alone or associated with macroscopic and microscopic changes, have been reported in obese subjects, possibly leading to malignant ventricular arrhythmias [13]. QRS fragmentation is a well-established marker of heterogeneous conduction and fibrosis in the myocardium in obese people, and it may also be associated with fatty infiltration and arrhythmogenic right ventricular dysplasia, where fragmented QRS is a diagnostic marker [14,56,57,58]. Furthermore, a higher occurrence of premature ventricular contractions is associated with the amount of epicardial adipose tissue, which correlates with the risk of ventricular tachycardia (VT)/ventricular fibrillation (VF) [14,59,60]. Moreover, a reduced heart rate variability (HRV) represents an autonomic dysfunction that may play a role in arrhythmogenesis [13,61]. Finally, QT prolongation has also been correlated with an increased BMI [13,62]. Thus, according to the latest evidence in cardiology and forensic medicine, the cause of death could potentially be attributed to extreme obesity alone. That being said, several cases of eosinophilic myocarditis in allergic people have been described in the scientific literature, but as ours is the first described case of eosinophilic infiltration of the SAN to date, it is not possible to establish whether an underlying allergy-mediated mechanism may be related to SCD [63,64]. Finally, the first case of fulminant necrotizing eosinophilic myocarditis after the first dose of the Pfizer-BioNTech mRNA COVID-19 vaccine was recently described, but in our case, it was not possible to assess the association with a drug reaction, as there were no data on COVID-19 vaccination status [65]. In conclusion, the autopsy failed to find the cause of death. In these cases, ESC guidelines and many authors recommend performing molecular autopsy [1,2,4,7,66,67,68,69]. In our case, during the molecular autopsy, we found a potentially deleterious variant: the intronic variant c.683+5G>A (rs397508122) in *KCNQ1* gene (NM_000218) (Table 1).

This mutation has already been described in more than 10 individuals with LQTS [33,34,35,36,37,70,71,72], as well as in a compound heterozygous case with other *KCNQ1* variants in a family with Jervell and Lange-Nielsen syndrome [41]. The c.683 + 5G > A variant was identified in a patient experiencing syncope triggered by emotion or swimming from the age of three years, while his father, also a carrier of the variant, was asymptomatic. In vitro studies showed a complete skipping of exon 4 and the resulting transcript encoded by the first 202 amino acids followed by 8 aberrant aminoacids and a premature stop codon [32]. As mentioned, following the current guidelines of the American College of Medical Genetics and Genomics and the Association for Molecular Pathology (ACMG/AMP), the variant c.683 + 5G > A was interpreted as likely pathogenic for LQTS [73]. In this case, the strongest indicator of pathogenicity is given by the fact that this variant is predicted to splice in gene *KCNQ1*, for which loss-of-function is a known mechanism of disease. *KCNQ1* encodes the α-subunit of the voltage-gated potassium channel responsible for the slow component of the delayed rectifier potassium current (IKs) [74]. Pathogenic variants involving *KCNQ1* result in a reduction in IKs, increasing the repolarization phase time of the action potential (QT interval), and are the most frequent cause of LQTS. Indeed, mutations in *KCNQ1*, *KCNH2* and *SCN5A* genes account for approximately 75% of clinical diagnoses of LQTS, while mutations in the remaining genes account for only 10% of LQTS cases. The inheritance pattern of pathogenic variants associated with *KCNQ1* gene mutations is both autosomal-dominant and recessive. Romano–Ward syndrome, Andersen–Tawil syndrome and Timothy syndrome (autosomal dominant forms) are characterized by the prolongation of the QT interval, sometimes associated with extracardiac manifestations, while Jervell and Lange-Nielsen syndrome (autosomal recessive form) also includes bilateral congenital deafness [2,75]. The variant c.683 + 5G > A identified in our index case on *KCNQ1* gene is an extremely rare variant in the general population, although present in more than ten LQTS cases in the literature. Although the in silico predictor tools are inconsistent between the predictions, Bozon et al. in 2012 published in vitro studies of this variant. The minigene splicing reporter assay using HeLa cells established a severe splicing alteration that results in a complete skipping of exon 4, resulting in a transcript that must be considered a null allele due to the nonsense mediated decay mechanism [32]. In conclusion, the genetic results and the circumstance of the death indicate that the SD was caused by LQTS. However, the presence of eosinophiles inside the SAN remains unexplained, while the significance of the abnormal heart weight and interstitial fibrosis is still uncertain. In our case, it is possible that eosinophilic infiltration could have triggered a malignant arrhythmia in a genetically predisposed heart. LQTS can correlate with heart structural anomalies (e.g., enlargement of the left atrium) [76,77], and has been rarely associated with microscopic features such as focal fibrosis and lipomatosis of the cardiac conduction system (CCS) and focal round cell ganglionitis of sympathetic trunks described in the scientific literature [78,79]. However, as mentioned, this is the first case in which an eosinophilic infiltrate of the SAN was found in a case of SD, and the potential correlation between this feature and LQTS is still to be explained. Moreover, as the woman had several risk factors for SCD (i.e., extreme obesity and smoking), a contribution of these factors cannot be ruled out. Therefore, it may be assumed that the SAN infiltration may have triggered a malignant arrhythmia in a genetically predisposed heart of an obese at-risk person, together contributing to the determinism of death.

## 4. Conclusions

Our case represents the challenging interpretation of SD cases, with a focus on the functional significance of nonspecific findings at autopsy and variants related to arrhythmic syndromes at postmortem genetic testing. Current evidence suggests that the observed microscopic abnormalities (i.e., interstitial fibrosis and eosinophilic inflammatory focus at SAN) are uncertain causes of SCD. Although several cases of unspecified mononuclear cell infiltration within the CCS are described in the scientific literature, the presence of an eosinophilic inflammatory focus of the SAN is a unique finding that has not yet been reported. Moreover, the discovery of a potentially deleterious variant associated with LQTS has made the determination of the etiology of the fatal arrhythmic event particularly challenging. Therefore, there were many anomalies that could have played a role in the causation of the sudden death, such as the extreme obesity, the cardiac anomalies and the *KNCQ1* variant. In our opinion, this case depicts the difficult interpretation of rare cardiac structural abnormalities in obese subjects carrying variants responsible for inherited arrhythmic disorders and the challenge for the forensic pathologist to make causal inferences in the determinism of SCD.

## Figures and Tables

**Table 1:** Report of rare genetic mutation identified in the index case.

Gene	*KCNQ1*
Isoform	NM_000218
Protein Variant	NA *
cDNA Variant	c.683 + 5G > A (rs397508122)
gnomAD Database	0.0002980%
ClinVar Database	2 Likely Pathogenic, 1 Pathogenic, 1 VUS
HGMD Database	CS072218
MaxEntScan (In Silico)	0
FSPLICE (In Silico)	1
GeneSplicer (In Silico)	0
ACMG KeyWords	PM2_Supp + PS4_Moderate + PS3_Strong
Classification	Likely Pathogenic in a LQTS context

* NA, Not Available.

## Data Availability

Not applicable.
